# Maternal minimum dietary diversity and associated factors among pregnant women, Southwest Ethiopia, 2021

**DOI:** 10.1186/s40795-021-00474-8

**Published:** 2021-10-28

**Authors:** Abel Girma Tilahun, Abebaw Molla Kebede

**Affiliations:** Department of Reproductive Health and Human Nutrition, College of Medicine and Health Science, Mizan-Tepi University, Mizan-Aman, Ethiopia

**Keywords:** Dietary diversity, Pregnant women, Kaffa zone, Southwest Ethiopia

## Abstract

**Background:**

Inadequate dietary diversity intake during pregnancy increases risks of intrauterine growth restriction, abortion, low birth weight, preterm birth, prenatal and infant mortality,and morbidity and has long-lasting health impacts. Dietary diversity during pregnancy promotes the health status of the mother and her fetus. This study aimed to assess the magnitude of minimum dietary diversity and associated factors among pregnant women attending antenatal care.

**Methods:**

A facility-based cross-sectional study was conducted among 274 pregnant women who attended antenatal care at Wacha primary hospital from January to February 2021. A systematic sampling method was used to select the study participants. The data were collected through face-to-face interviews using a structured and semi-structured questionnaire. Bivariate logistic regression was done to identify factors associated with maternal dietary diversity. Finally, multivariate logistic regression was done, and variables that showed *P* values of < 0.05 were considered statistically significant.

**Result:**

The magnitude of minimum dietary diversity was 51% (95% CI: 44.5, 56.7). The mean (±SD) minimum dietary diversity score was 4.5 (± 1.268) with a minimum of 1 anda maximum of 8 food groups consumed out of ten food groups. Age fewer than 25 years (AOR 4.649; 95% CI; 1.404, 15.396), and the age group between 25 to 34 years (AOR 3.624; 95% CI: 1.315, 10.269), husband age group of 26 to 34 years (AOR 2.238; 95% CI; 1.028,4.873), and 35 to 44 years (AOR 3.555; 95% CI; 1.228,10.292) and nutrition awareness of women (AOR 2.182; 95% CI; 1.243, 3.829) were significantly associated with minimum dietary diversity.

**Conclusion:**

The consumption of minimum dietary diversity of the pregnant mothers was found to be low. Women aged less than 25 and age between 25 to 34 years, husband’s age between 26 to 34 and 35 to 44 years, and nutrition awareness were the factors significantly associated with minimum dietary diversity. Therefore, providing nutrition education and counseling service warranted to promote maternal dietary diversity.

## Background

Poor dietary diversity is the major public health concern for pregnant women in resource-poor environments all over the world [1, 2]. Dietary diversity is the number of food groups consumed across and within food groups over a given reference period [1, 3]. It is a key dimension of diet quality which is defined as a diet that delivers adequate amounts of selected micronutrients, to meet the needs of women of reproductive age and the indicators of nutritional adequacy [1, 3]. Pregnancy by itself places a further burden on women’s nutritional requirement which increase the nutrient needs to meet the demands of both the mother and the developing fetus [4]. Dietary diversity in pregnant women is used to reduce maternal mortality and morbidity and it is also a foundation to the developing fetus’ growth and reduced perinatal outcome complications [5, 6]. In contrast suboptimal diets of pregnant women further impacts the health of the mother, the developing fetus, and the newborn [7]. Furthermore, inadequate dietary diversity intake during pregnancy results in increased risks such as maternal anemia, intrauterine growth restriction (IUGR), abortion, low birth weight (LBW), preterm birth, prenatal and infant mortality, and morbidity and increase the risk of chronic disease in the later period of life [8–13].

Poor dietary quality is one of the leading causes of premature death and diseases globally [14]. Maternal inadequate dietary diversity contributed to 7% of the global disease burden and at least one-fifth of maternal deaths and poor maternal outcomes [15, 16] and while one million neonates die on the first day and in the first week of life, as linked with increasing trends in maternal anemia, mortality, and adverse birth outcomes [17]. In Sub-Saharan Africa, the burden of nutritional deficiency among pregnant women is still high [18–20]. Micronutrient deficiency and maternal undernutrition can affect pregnancy birth outcomes, increase maternal mortality and reduce economic outcomes [9, 21–23]. In Ethiopia, the maternal mortality ratio is 401 per 100,000 live birth and while the neonatal mortality rate is 28 per 1000 live births [23] and while about 22% of women are thin, 24% anemic and 8% of women are obese [24] and 23 and 15% of women reproductive age in South Nation, Nationality and People Region (SNNPR) of Ethiopia are anemic and thin due to inadequate dietary intake respectively [25]. In resource-poor environments across the globe, there are dominant plant-based staple foods and diets that lack vegetable, fruits, and animal-source foods [1]. There is low minimum dietary diversity in Ethiopia among pregnant women [26]. Only 47% of pregnant women had to meet minimum dietary diversity in Ethiopia at the national level [27] and 28% of pregnant women had to meet adequate dietary diversity in the SNNPR of Ethiopia [22] where on an average 3.7 of 10 food groups were eaten by pregnant women [23].

To adequately provide the required nutrients and to reduce maternal malnutrition one of the best-recommended strategies among pregnant women is dietary diversification through increasing food group consumption in daily diet [28]. The government of Ethiopia has developed the second phase National Nutrition Program (NNP II 2016–2020), which focuses on the critical 1000-day window – from pregnancy through the first two years of life – to address malnutrition and with one of its program objectives of improving pregnant and lactating women’s nutrition through comprehensive and routine nutritional assessments and counseling services [23].. The main identified factors for poor dietary diversity in developing countries were socio-demographic and economic factors, food insecurity, lack of nutrition counseling and environmental factors [17, 27, 29–35]. Identifying and addressing the possible factors associated with maternal dietary diversity has a significant contribution to enhancing the nutritional and health status of both mother and her fetus. The finding of this study will help policymakers and planners to set their target with interventions in the study area and might be a means of achieving the sustainable development goals (SDGs) in an integrated manner. However, there is a lack of information in this study area. Therefore, this study was aimed to assess maternal dietary diversity and associated factors among pregnant women attending antenatal care (ANC) at Wacha primary hospital, southwest asia Ethiopia.

## Methods

### Study area

The study was conducted at Wacha primary hospital which was established in 2005. It is found in Chena town, Kaffa Zone, Southwest Ethiopia which is located 541 km far from Addis Ababa, the capital city of Ethiopia and it is the only hospital in Chena district. The hospital has been giving health services for the total population of 30,891 people in its catchment area with different four major departments including medical, pediatrics, surgical, and obstetrics, and gynecology. It also provides outpatient service, ophthalmology, emergency, antiretroviral Therapy (ART) clinic, and ANC clinic. There were a total of 723 pregnant women who had attended ANC in 2020/21 and on average around 30–40 followers have been attended ANC clinic every day at Wacha primary hospital.

### Study design and study period

A facility-based cross-sectional study was conducted from January to February 2021.

#### Source population

All pregnant women attended the ANC clinic at Wacha primary hospital, Kaffa zone.

#### Study population

All randomly selected pregnant women attending ANC clinic at Wacha primary hospital from 16 weeks gestational age were included in the sample.

### Sample size determination

The required sample size was determined using single population proportion formula based on the assumption of high minimum dietary diversity of 55.2% in Bale zone, Ethiopia [33], 5% margin of error, 95% of Confidence Interval (CI), 10% of non-response rate, and by using the correction formula since the target population of the study area was below 10,000. Then the final sample sizes 274 of pregnant women were included.

### Sampling method

The study participants were selected by using a systematic random sampling technique. The sampling interval was determined by calculating monthly average attendance for ANC follow-up divided by the required sample size, and then, the first study participant was selected randomly and then every third pregnant woman was included.

#### Inclusion criteria

Pregnant women with greater than 16 week’s gestational age and who lived in the study area for at least one year were included in the study.

#### Exclusion criteria

Pregnant women who were unable to speak&/hear and who have seriously ill during data collection.

### Study variables

The dependent variable of this study was dietary diversity status, and independent variables like age, marital status, occupation, residence, educational status, family size, household head, nutrition awareness of women, maternal health status, husband support, income, having mobile phone, radio and bank account, garden, water source, availability of latrine, marketing, and household food security and nutritional status of pregnant women.

### Data collection technique

The data were collected through face-to-face interviews using pretested structured and semi-structured questionnaires adapted from different kinds of literature [2, 33, 36]. The data were collected by well-trained Nurse professionals. The questionnaire had three parts. The first part includes socio-demographic factors (age, marital status, residence, family size, education, occupation, and others). The second part was dietary-related information questionnaires which were adapted and modified from the Food and Agriculture Organization of the United Nations (FAO) 2016 [36]. The dietary diversity questioner has ten different food groups based on their nutrients:1) grains, white root, tubers, and plantains, 2) pulses (beans, peas, and lentils), 3) nuts and seeds, 4) dairy, 5) meat and fish (poultry and fish), 6) eggs, 7) dark green leafy vegetables, 8) vitamin A-rich fruits and vegetables, 9) others vegetables, and 10) others fruits. It was assessed by using 24-h open dietary recall methods; one point was given to each food group consumed over the past 24 h before the survey period. The participants were asked about all food and beverage consumed during the day and night including any snack in the past 24 h and the interviewer were probing for any food types forgotten by participants. Each food or beverage that the respondent mentions was circled underlined on a predefined list. The foods not included on the predefined list were classify by the principal investigator on an existing predefined food group or recorded in a separate place on the questionnaire and coded and organized later into one of the predefined food groups [36].

The third part was household food security status which was assessed by using Household Food Insecurity Access Scale (HFIAS) [37]. The household food security was categorized as food insecure for those who score 2 and above out of 27 household food insecurity indicators and while food secures categorized for pregnant women who scored less than 2 out of 27 household food insecurity indicators.

Nutritional status of pregnant women was assessed by using mid-upper arm circumference (MUAC) which was measured halfway between the olecranon process and acromion process by using -non-stretchable tape to the nearest 0.1 cm. Nutritional status was defined as MUAC less than 22 cm were undernutrition and while 22 cm or more was considered to be normal nutritional status [38, 39].

### Data quality control

Initially, a survey questionnaire was prepared in English translated to the local language and translated back to English to check for consistency. A three days training was given for data collectors and supervisors and a pretest was done on 5% of the study sample size. The Cronbach’s alpha was calculated (*P* = 0.78). The data were checked by the principal investigator on the daily basis for completeness and consistency.

### Data processing and analysis

After the data were cleaned and coded, the data were entered in Epidata 3.1 version software and exported to statistical package for social science (SPSS) version 21 for analysis. A minimum dietary diversity score (MDDS) was dichotomized as meet minimum dietary diversity for pregnant women who consumed 5 and above out of ten food groups coded as 1 and while unmeet minimum dietary diversity for those who consume less than 5 out of ten food groups in the past 24 h coded as 0. Descriptive analysis was done to calculate the mean, frequencies, and percentage distributions for the variables. A stepwise backward elimination logistic regression was done to identify variables associated with minimum dietary diversity. The model fitness was checked by using Hosmer and Lemeshow statistic (*P* = 0.47), which showed the model was fitted. Bivariate logistic regression was done to identify the covariate associated with minimum dietary diversity and independents variables and the variables with a *p*-value less than 0.05 were considered for multivariable logistic regression to control all possible confounders and to determine the strength of association between minimum dietary diversity and each explanatory variable. The strength of association was measured with an adjusted odds ratio (AOR) with a 95% CI. Finally, the variables with a *P*-value less than 0.05 were considered statistically significant.

## Result

### Socio-demographic and other related characteristics of pregnant women at Wacha primary hospital, Southwest Ethiopia, 2021 (*N* = 263)

A total of 274 respondents were participated in this study making a response rate of 96%. The mean (±Standard deviation(SD)) age of the respondents was 26.2 (±5.48) years and more than half of (52.1%) women were in the age group of < 25 years. Nearly half of the respondents (47.5%) were protestant religion followers, and the majority of participants (96.2%) were married. Around 32.3% of pregnant women were attended primary school by their educational status. Regarding the occupation 53.2% of pregnant women were housewives, and the majority of (76.4%) pregnant women were Kaffa in ethnicity and greater than half (59.7%) of the respondents were urban residents. About 64.3% of participants had a family size of < 5; and while 73.4% of pregnant women’s households heads were husbands. The majority of (97.7%) of pregnant women were free from any illness for the past 2 weeks before the data collection and more than half (66.6%) of pregnant women were food insecurity. Around 30.8% (95%CI: 25.1, 36.5) of pregnant women were undernourished and while the large majority (69.2, 95% CI: 63.5, 74.9) were had normal nutritional status (Table 1).

**Table 1:** Socio-demographic characteristics of pregnant women at Wacha primary hospital, Southwest Ethiopia, 2021 (*N* = 263)

Variables		Frequency	Percentage
Age of women	< 25	137	52.1%
	25–34	97	36.9%
	> 34	29	11.0%
Age of husband	19–25	45	17.1%
	26–34	134	51%
	35–44	84	31.9%
Marital status	Married	253	96.2%
	Single	5	1.9%
	Widowed	2	0.8%
	Divorced	3	1.1%
Women religion	Orthodox	113	43%
	Protestant	125	47.5%
	Muslim	20	7.6%
	Other *	5	1.9%
Residence	Urban	157	59.7%
	Rural	106	40.3%
Ethnicity	Kaffa	201	76.4%
	Amhara	39	14.8%
	Other**	23	8.8%
women education	Unable to read and write	77	29.3%
	Able to read and write	12	4.6%
	primary	85	32.3%
	secondary	43	16.3%
	college and above	46	17.5%
husband educational	Unable to read and write	62	23.6%
	Read and write	9	3.4%
	Primary (1–8)	65	24.7%
	Secondary (9–12)	75	28.5%
	College and above	52	19.8%
Women occupational	Housewife	140	53.2%
	Government employee	52	19.8%
	Merchant	34	12.9%
	Daily labor	37	14.1%
Head of household	Husband	193	73.4%
	Wife	25	9.5%
	Both	45	17.1%
Family size	< 5	169	64.3%
	> = 5	94	35.8%
Income	<=1500	123	46.8%
	> 1500	140	53.2%
Shopping	Wife	240	91.3%
	Husband	21	8%
	Other	2	0.8%
Nutrition awareness	Yes	158	60.1%
	No	105	39.9%
Husband support	Yes	244	92.8%
	No	19	7.2%
Bank account	Yes	99	37.6%
	No	164	62.4%
Mobile	Yes	145	55.1%
	No	118	44.9%
Radio	Yes	156	59.3%
	No	107	40.7%
Garden	Yes	184	70%
	No	79	30%
Fasting	Yes	84	31.9%
	No	179	68.1%
Latrine	Yes	262	99.6%
	No	1	0.4%
Water source	Tap	47	17.9%
	Spring	170	64.6%
	Well	46	17.5%
Illness	Yes	6	2.3
	No	257	97.7%
Food security status	< 2	175	66.6%
	≥ 2	88	33.4%
Nutritional status	< 22	81	30.8%
	≥ 22	182	69.2%

Notes: N = total number of participants, * Catholic, Pagan, ** Bench, Oromo, Wolayita, Tigre, Selte

### Food consumption patterns of pregnant women at Wacha primary hospital, Southwest Ethiopia, 2021 (*N* = 263)

**Fig. 1:**
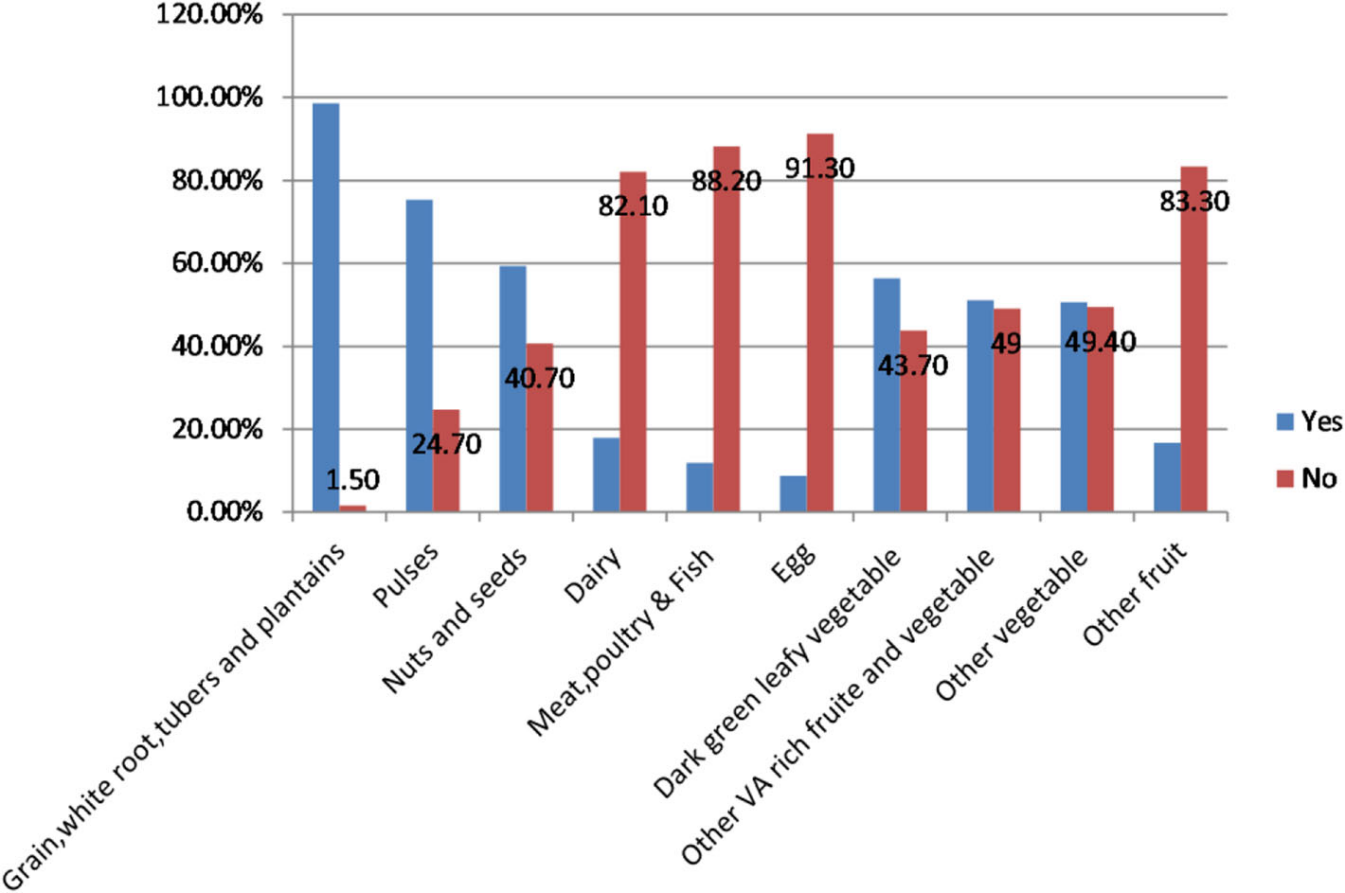
Food group’s consumption patterns of pregnant women at Wacha primary hospital, Southwest Ethiopia 2021

Regarding the food groups consumed by pregnant women in the previous 24 h, about 98.5% consumed starchy stable food groups, 75.3% consumed pulses, food groups, 56.3% were consumed dark green leafy vegetables and 51% were consumed other vitamin –A-rich fruits and vegetables and while the least consumed food groups were eggs (8.7%) and meat and fish (11.8%) (Fig. 1).

### Maternal minimum dietary diversity among pregnant women at Wacha primary hospital, Southwest Ethiopia, 2021, (*N* = 263)

According to this study, out of the 10 food groups that the mean dietary diversity score among pregnant women was 4.5 ± 1.268 SD with scores ranging from 1 to 8 food groups. About 51% (95% CI: 44.5, 56.7) of pregnant women meet the minimum dietary diversity scores, and while around 49% (95% CI: 43.3, 55.5) were consumed less than five food groups (unmeet minimum dietary diversity score).

### Factors associated with minimum dietary diversity

In bi-variable logistic regression analysis the variables; women occupation being employee, nutrition awareness, head of household being husband, age of women fewer than 25 and age between 25 to 34 years and age of husbands 26 to 34 years were significantly associated (*P*-value less than 0.05) with the minimum dietary diversity score. In multivariable logistic regression analysis, the variables; the age of women fewer than 25 and age between 25 to 34 years, age of husbands between 26 to 34 years and age group 35 to 44 years, and maternal nutritional awareness were significantly associated with minimum dietary diversity score. The pregnant women with the age group of fewer than 25 years were 4.64 times more likely (AOR = 4.65; 95% CI; 1.404, 15.396) and in the age group of 25 to 34 years were 3.62 times more likely (AOR = 3.624; 95% CI: 1.315, 10.269) to have minimum dietary diversity score compared with than those who had aged greater than 34 years. Women having husbands age group 26 to 34 years were 2.2 times more likely (AOR = 2.238; 95% CI; 1.028,4.873) and age group 35 to 44 years were 3.555 times more likely (AOR = 3.555; 95% CI; 1.228,10.292) to have minimum dietary diversity score than those who having husband age group between 19 to 25 years olds. The pregnant women who received nutrition information during pregnancy were 2.2 times more likely (AOR = 2.182; 95% CI; 1.243, 3.829) to meet minimum dietary diversity score than those who didn’t get nutrition information (Table 2).

**Table 2:** Factors associated with minimum dietary diversity at Wacha primary Hospital, Southwest Ethiopia, 2021 (N = 263)

Variable		Dietary diversity		COR (95%CI)	AOR (95%CI)
		Poor	Good		
Age of women	< 25	65	72	2.460 (1.047,5.789)	4.65 (1.404,15.396)*
	25–34	45	52	2.57 (1.063,6.204)	3.62 (1.315,10.269)*
	> 34	20	9	1	1
Age of husband	19–25	29	16	1	1
	26–34	60	74	2.24 (1.111,4.497)	2.24 (1.028,4.873)*
	35–44	41	43	1.90 (0.902,4.006)	3.55 (1.228,10.292)*
Occupation of women	Housewives	63	77	1	1
	Employee	32	20	0.510 (0.267,0.980)*	0.61 (0.268,1.383)
	Merchant	17	17	0.39 (0.386,1.732)	0.90 (0.408,2.135)
	Daily laborer	18	19	0.864 (0.418,1.732)	1.310 (0.555,3.091)
House hold head	Husband	89	104	2.12 (1.081,4.151)*	1.049 (0.406,2.706)
	Wife	12	13	1.964 (0.727,5.386)	0.598 (0.261,1.371)
	Both	29	16	1	1
Nutrition awareness	Yes	70	88	1.676 (1.019,2.758)	2.182 (1.243,3.829)*
	No	60	45	1	1

Notes: * statistically significant at p-value less than 0.05

## Discussion

### Minimum dietary diversity of pregnant women

This study was aimed to assess the minimum dietary diversity and associated factors among pregnant women attending ANC at Wacha primary hospital, Southwest Ethiopia. According to this study finding around 51% of pregnant women had met a minimum dietary diversity which is lower than the study finding from Burkina Faso (68.4%) [30], Addis Ababa (60.9%) [40], Hosanna town of Ethiopia (75.8%) [41], and Alamata (61.2%) [2]; and while higher than the national level of Ethiopia (47%) [27], SNNPR region of Ethiopia (28%) [23], East Gojjam Zone of Northwest Ethiopia (45%) [42], Jille Tumuga (31.4%) [32], Bale (44.8%) [33], Dire Daw city (43%) [39] and Shashemene town (25.4%) [43]. The possible explanation for this prevalence discrepancy might be due to study periods differences, geographical differences, socio-cultural differences, and differences in the measurement of dietary diversity which some study uses nine food groups and while other studies used the 14 food groups, and those consumed four or more food groups of the fourteen food groups were classified as minimum dietary diversity which will result in a greater score than this finding.

### Food groups consumption patterns of pregnant women

In this study about 98.5 and 75.3% of pregnant women consumed grain, white roots, tuber, and plantains food groups (pasta, taro, enset, potato; teff, wheat, maize, rice in the form of injera, bread, porridge) and pulse food groups (beans, peas, and lentils) respectively and while the least consumed food groups were eggs (8.7%), meat, poultry and fish food groups (11.8%), other fruits (16.7%) and dairy food groups (17.9%) in the previous 24 h. it is consistent with the others study findings from Kenya [31], Hossana Town of southern Ethiopia [41], SNNPR of Ethiopia [25], and Bale zone of southeast Ethiopia [33]. Moreover, it is consistent with many others studies finding from developing countries where their main dietary sources are cereal-based food groups [44]. But there is a slight difference from the study conducted in the East Gojjam zone where the commonly consumed dietary groups were legumes, nuts, and seeds (85.5%) followed by starchy staples (64.7%) [42]. This finding was also inconsistent with the study finding from the SNNPR regional state of Ethiopia where 71% vs.56.3% of pregnant women were consumed vitamin A-rich dark green leafy vegetables and 19% vs. 51% of pregnant women were consumed other vitamin A-rich fruits and vegetables food groups [25]. This inconsistency finding could happen due to seasonal variation, self-reporting differences, and the measurement difference of dietary diversity.

### Factors associated with minimum dietary diversity of pregnant women

The pregnant women with the age group of fewer than 25 years were 4.65 times more likely to attain the minimum dietary diversity score than those with the age group greater than 34 years old. This could be due to the younger pregnant women might know that the double burden of malnutrition during younger age so that they might be trying to have adequate dietary diversity during their pregnancy. Pregnant women with the age group of 25 to 34 years were 3.624 times more likely to have the minimum dietary diversity score than those with the age group greater than 34 years. This finding is consistent with the study conducted in Eat Gojjam zone where maternal age was significantly associated with the dietary diversity of pregnant women in bi-variable logistic regression as the age of the mother increases the likelihood of inadequate dietary diversity was increased [42]. This might indicate that as the women age becomes old their access to get adequate dietary diversity might become poor.

The pregnant women with their husband’s age group is 26 to 34 years old were 2.20 times more likely to have minimum dietary diversity than those with their husband age group is 19 to 25 years olds. Besides, the pregnant women with their husband’s age group are 35 to 44 years were 3.56 times more likely to have minimum dietary diversity than those with their husband age group were 19 to 25 years olds. The possible explanation for this could be because the women who have older age husbands might support the pregnant women to have adequate dietary diversity during pregnancy than the women who have younger age husbands.

The pregnant women who had received nutrition awareness during pregnancy were 2.182 times more likely to have a minimum dietary diversity score than those who had not received nutrition awareness during pregnancy. This finding is consistent with the finding from Ethiopia where lack of counseling about dietary diversity was one of the factors significantly associated with inadequate dietary diversity in Ethiopia [27]. It is also consistent with the study conducted in Addis Ababa where having nutritional information during pregnancy was significantly associated with minimum dietary diversity [40].

This study cannot make the causal relationship (difficult to know which precedes the exposure or outcome). Hence, it is a cross-sectional study. The study is unable to generalize to the population because of the facility-based nature of this study. In addition, this study might not give the exact figure of the dietary diversity practice due to a recall bias and is self-reported. A relatively small sample size of this study might be the other limitation.

## Conclusion

This study has shown that the overall consumption of adequate dietary diversity of pregnant mothers was found low. The variables like women of age fewer than 25 and age between 25 to 34 years, husbands age between 26 to 34 and 35 to 44 years, and maternal nutrition awareness were positively significantly associated with adequate dietary diversity of pregnant mothers at Wacha primary hospitals. Therefore, providing nutrition education and counseling services is warranted to promote maternal dietary diversity. Moreover, other researchers should conduct further study to see the association of husbands ages between 26 to 34 and 35 to 44 years and mothers ages fewer than 25 and age between 25 to 34 years with minimum dietary diversity during pregnancy. Encourage pregnant mothers to improve their dietary diversity by increasing the daily consumption of food groups like animals source foods and different types of fruits.

## Data Availability

The datasets used and or analyzed during the current study are available from the corresponding author on reasonable request.

## Abbreviations

ANC: Antenatal Care
AOR: Adjusted Odds Ratio
CI: Confidence Interval
FAO: Food and Agriculture Organization
FANTA: Food and Nutrition Technical Assistance
HFIAS: Household Food Insecurity Access Scale
MDDS: Minimum Dietary Diversity Score
MUAC: Mid upper arm circumference
SD: Standard Deviation
SNNPR: South Nation, Nationality and People Region
SPSS: Statistical Package for Social Science
WHO: World Health Organization
